# Melanoma and the Unfolded Protein Response

**DOI:** 10.3390/cancers8030030

**Published:** 2016-02-27

**Authors:** Erin K. Sykes, Swetlana Mactier, Richard I. Christopherson

**Affiliations:** School of Molecular Bioscience, The University of Sydney, Darlington 2006, Australia; swetlana.mactier@sydney.edu.au (S.M.); richard.christopherson@sydney.edu.au (R.I.C.)

**Keywords:** melanoma, unfolded protein response, UPR, ER stress

## Abstract

The UPR (unfolded protein response) has been identified as a key factor in the progression and metastasis of cancers, notably melanoma. Several mediators of the UPR are upregulated in cancers, e.g., high levels of GRP78 (glucose-regulator protein 78 kDa) correlate with progression and poor outcome in melanoma patients. The proliferative burden of cancer induces stress and activates several cellular stress responses. The UPR is a tightly orchestrated stress response that is activated upon the accumulation of unfolded proteins within the ER (endoplasmic reticulum). The UPR is designed to mediate two conflicting outcomtes, recovery and apoptosis. As a result, the UPR initiates a widespread signaling cascade to return the cell to homeostasis and failing to achieve cellular recovery, initiates UPR-induced apoptosis. There is evidence that ER stress and subsequently the UPR promote tumourigenesis and metastasis. The complete role of the UPR has yet to be defined. Understanding how the UPR allows for adaption to stress and thereby assists in cancer progression is important in defining an archetype of melanoma pathology. In addition, elucidation of the mechanisms of the UPR may lead to development of effective treatments of metastatic melanoma.

## 1. The Unfolded Protein Response

**Figure 1:**
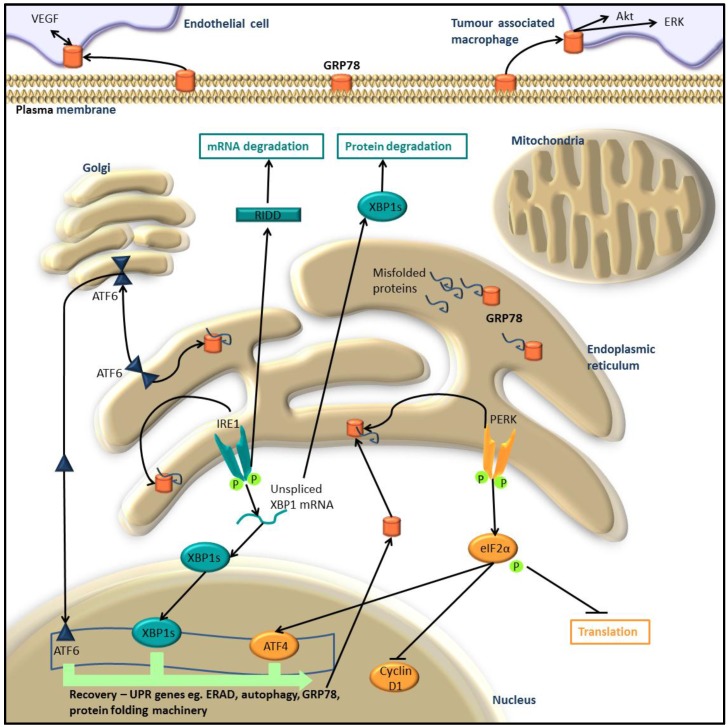
Cellular recovery modulated by the UPR signalling cascade. Misfolded proteins within the ER are bound by the ER chaperone GRP78, resulting in its displacement from 3 ER transmembrane proteins. The 3 proteins PERK, IRE1 and ATF6, initiate the UPR signalling cascades. PERK dimerises, trans-autophosphorylates then phosphorylates eIF2α. Active eIF2α then inhibits cyclin D1 to halt the cell cycle, preventing Met-tRNA recruitment to the 40S ribosomal subunit for global suppression of protein synthesis, eIF2α also activates the transcription factor ATF4. IRE1 dimerises and trans-autophosphorylates to become active, cleaving unspliced XBP1 mRNA through its ribonuclease activity to form an active transcription factor, XBP1s. XBP1 splicing also results in protein degration via activation of ERAD and autophagy. ATF6 freed from GRP78, translocates to the Golgi where its cytosolic-transcription factor domain is cleaved by SiP1 and SiP2, then localises to the nucleus. The 3 transcription factors ATF4, XBP1s and ATF6 increase the expression of UPR responsive genes to maintain homeostasis, including ER-chaperones, ERAD and autophagic proteins. The chaperone GRP78 is also upregulated, moving to the cell surface and into circulation. Circulting GRP78 propogates growth in tumour associated macrophages and endothelial cells by activating signallling cascades with in these cells.

Membrane and secretory proteins, that account for 30% of human proteins [1], are folded and mature within the endoplasmic reticulum (ER) before export to the cell surface via the trans-Golgi network. Due to the frequent bulk of protein processing occurring within this organelle, the ER has an exquisitely fine-tuned stress response to cope with the protein load. The UPR is activated by the accumulation of unfolded protein within the ER that initiates a widespread but refined signaling cascade (Figure 1). 

### 1.1. Activation of the UPR

The UPR signaling cascade is mediated by three key ER trans-membrane proteins, PERK (eukaryotic translation initiation factor 2-alpha kinase 3), IRE1 (serine/threonine-protein kinase/endoribonuclease IRE1) and ATF6 (activating transcription factor 6). These proteins reside within the ER bound via their luminal domains to a regulatory protein GRP78 (glucose-regulated protein78 kDa), which is considered the master regulator of the UPR [2]. GRP78 is a member of the heat shock 70 protein (HSP70) family of chaperones. During stress when unfolded proteins accumulate in the ER, GRP78 binds the unfolded proteins, releasing the three UPR mediators. Each induces a distinct signal transduction pathway mediating a particular arm of the UPR. The three arms of the UPR cumulatively result in up-regulation of ER resident chaperones, the suppression of global protein synthesis and the degradation of existing proteins via ER-associated degradation (ERAD) and degradation of organelles through autophagy. 

The release of IRE1 from GRP78 allows it to dimerise and undergo auto-phosphorylation resulting in activation of its RNase (ribonuclease) activity [3,4]. The activated IRE1 RNase domain then cleaves the mRNA transcript of XBP1 (X-box binding protein 1) [5]. The now mature XBP1 mRNA is translated into a functional transcriptional factor, XBP1s (XBP1 spliced) (Figure 1). 

PERK acts similarly to IRE1 upon GRP78 release, allowing it to dimerise and phosphorylate eIF2α (eukaryotic initiation factor 2α subunit) to suppress global protein translation [4]. Phosphorylated eIF2α also activates ATF4 to facilitate transcription of UPR responsive genes (Figure 1) [6]. Additionally, ATF4 initiates negative feedback on eIF2α, by promoting expression of another transcription factor CHOP (C/EBP homologous protein) that de-phosphorylates eIF2α via GADD34 (Growth arrest and DNA damage-inducible protein) and PP1(Protein phosphatase 1) [7,8].

ATF6 exists in two isoforms ATF6α and ATF6β, that upon GRP78 release, translocate to the Golgi compartment where they are cleaved by the proteases SiP1 and SiP2, releasing the ATF6 cytosolic domain [9,10]. The ATF6 domain translocates to the nucleus where it acts via ERSE (cis-acting ER stress response element) on the promoters of several ER chaperone genes, up-regulating their transcription [11]. 

### 1.2. Return to Homeostasis

In order to repeal stress, the UPR reduces the influx of proteins into the ER. IRE1 phosphorylation of eIF2α prevents Met-tRNA (methionine-transfer RNA) recruitment and results in global suppression of protein synthesis, reducing the protein folding burden of the ER [12]. eIF2α mediates the recruitment of Met-tRNA to the 40S ribosomal subunit, and is the rate-limiting step in protein translation [13]. Selected UPR genes are preferentially translated via cap-independent translation of internal ribosome-entry sites [14]. Additionally, active IRE1 selectively degrades mRNA bound for the ER in a process called RIDD (regulated IRE1-dependent decay). In mammals, mRNA with a conserved sequence similar to that found in the transcription factor XBP1, are targeted by IRE1 for degradation, thereby relieving the protein processing load in the ER [5]. All three mediators of the UPR; ATF6 directly, IRE1 via XBP1s and PERK via ATF4, act to increase the expression of several chaperones, including GRP78, to assist in protein folding within the ER [11,15,16,17]. Other non-stress specific responses are also perturbed by these transcription factors such as amino acid metabolism, redox state and mitochondrial metabolism [18,19,20,21]. 

The overlap that exists between the signaling cascades is potentially a means by which increased control of the UPR response and its outcome can be exerted. In lower eukaryotes such as yeast, the entire UPR is mediated by IRE1, however higher eukaryotes have adapted to include two additional UPR cascades, allowing for more precise control of this stress response [22]. During ER stress, these three arms of the UPR act in concert to return the cell to homeostasis [23]. There is considerable overlap between the three initial signaling cascades presumably to enable fine-tuning of the UPR to adapt to different levels of stress within the cell, and control the result of the UPR. 

The UPR also controls two protein and organelle degradative pathways, ERAD (ER associated degradation) and autophagy (Figure 1) that are responsible for the clearance of aberrant proteins from the cell. The induction of ERAD mainly occurs through IRE1-XBP1s signaling [4]. ERAD is another way in which the ER controls homeostasis through selective degradation via the destruction of misfolded proteins present in the ER. During ER stress, the induction of the UPR expands the capacity of ERAD to eliminate unfolded proteins. Several ERAD components are up-regulated through UPR transcription factors (Figure 1), while ER chaperones up-regulated through the UPR, selectively target misfolded proteins to the cytoplasm for poly-ubiquitination and degradation by the 26S proteasome [24]. ERAD and the UPR exhibit reciprocal activation, acting in concert to clear misfolded proteins from the ER [24]. Disruption of ERAD via proteasome inhibition has been shown to induce cell death in cells with ER-stress, as such it is concluded that ERAD is crucial in adaption to chronic UPR and the avoidance of melanoma to UPR-induced apoptosis [25,26,27]. Activation of the UPR in turn activates a co-operative mechanism within the cell known as autophagy. Autophagy is the process of degrading and recycling whole organelles via autophagosomes, a membrane vesicle that targets its package to lysosomes. The UPR co-activates the autophagy program via both PERK-eIF2-ATF4 and JNK signaling [6,28]. Similar to the UPR, autophagy promotes cellular recovery by degrading proteins in cancerous cells, while resulting in cell death in un-transformed cells [28,29]. For the cell to process the misfolded proteins, the UPR prompts ER expansion. Autophagy is instrumental therefore in the resolution of the UPR by degrading excess organelles whose protein folding capacity is no longer required. When autophagy is inhibited in cells under acute ER stress, the cells are unable to recover and undergo apoptosis [30], suggesting that autophagy is an essential aspect of the UPR program, enabling avoidance of UPR-induced apoptosis in cancers. Patients with metastatic melanoma with high levels of autophagy had shorter survival and exhibited less response to temozolomide, a DNA damaging agent, and sorafenib, a RAF inhibitor [31]. The implications of autophagy in cancer progression are extensive and are well reviewed by Mathew *et al.* and White [32,33]. 

Collectively the UPR relieves ER stress and returns cells to homeostasis through a cooperative, highly co-ordinated response involving inhibition of global protein synthesis, up-regulation of UPR-responsive genes involved in ER protein folding and through the selective degradation of ER-targeted mRNA by RIDD, misfolded proteins via ERAD and whole organelles/proteins by autophagy. 

### 1.3. UPR-Induced Apoptosis

In the case of acute or prolonged ER stress when the cell fails to return to homeostasis, the UPR can induce apoptosis. UPR-induced apoptosis is initiated through the same signaling mechanisms that are triggered to restore the cell to homeostasis and, as such, the UPR engages in a fine balancing act between cellular recovery and death. This is achieved through complex regulation in which the three UPR arms modulate one another to promote either survival or death, and in the case of cancer the UPR encourages cellular recovery as the outcome.

**Figure 2:**
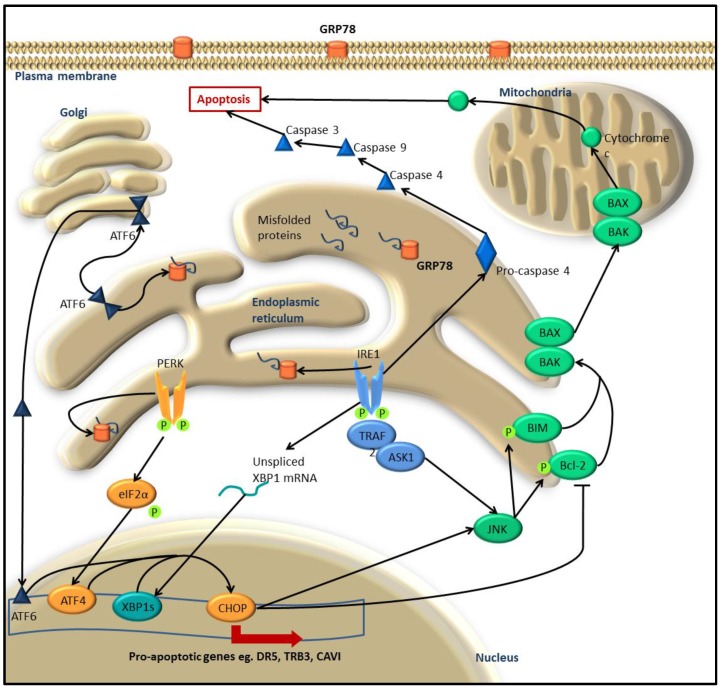
Apoptosis modulated by the UPR signalling cascade. In the case of acute, prolonged ER-stress, the UPR stimulates apoptosis modulated by the same 3 proteins that initiate UPR cellular recovery. Activated PERK, IRE1 and ATF6 increase the expression of the transcription factor CHOP. CHOP up-regulates several pro-apoptotic genes including DR5 (death receptor 5), TRB3 (tribbles homolog 3) and CAVI (carbonic anahydrase VI). Additionally, CHOP activates JNK (c-JUN N-terminal kinase) that propagates apoptosis by phosporylating Bcl-2 (B-Cell CLL/Lymphoma 2) and BIM (Bcl2-like protein 11) to initiate Bcl-2 apoptotic signalling and release of cytochrome C. JNK is also activated by dimerised IRE1 through TRAF2-ASK1 signalling. Additionally, IRE1 directly cleaves procaspase-4 to initiate the apoptotic caspase cascade.

On failing to resolve ER-stress, UPR-induced apoptosis is activated by both PERK and IRE1 cascades and the direct activation of caspase-12. Prolonged ER stress leads to PERK phosphorylating eIF2α and inducing ATF4 expression that in turns results in the up-regulation of CHOP, a transcription factor that stimulates the expression of several pro-apoptotic genes (Figure 2) [34,35]. Increased expression of CHOP by the UPR results in decreased Bcl-2 (apoptosis regulator Bcl-2) levels and translocation of Bax (Bcl-2 antagonist of cell death) to the mitochondria to induce apoptosis [36]. In this way, the UPR is able to mediate apoptosis through well characterised apoptotic signaling pathways that result in mitochondrial membrane disruption. Acute ER-stress and activation of IRE1 signaling can relocalise Bak and Bax (Bcl-2 antagonist of cell death) to the mitochondria to propagate apoptosis [37]. Activation of IRE1 in response to prolonged ER-stress will induce apoptosis via recruitment and phosphorylation of TRAF2 (TNF-associated receptor factor 2) that activates JNK through the ASK1 signaling cascade (Figure 2) [38]. Protein kinase JNK, then promotes mitochondrial-dependent apoptosis involving unknown downstream targets. Caspase-12 (human ortholog caspase-4) is itself a critical effector in UPR apoptosis, indeed null caspase-12 mutants have reduced sensitivity to ER-stress induced apoptosis [39]. Pro-caspase-12 resides in the ER-membrane and when prolonged, acute ER-stress is present, phosphorylated IRE1 cleaves caspase-12 initiating the caspase cascade cleaving caspase-9 then caspase-3, eventuating in apoptosis [40]. The ability to bypass classical apoptotic cascades is of particular interest for cancer research. Oncogenic mutations render the cells resistant to apoptotic mechanisms, therefore this particular UPR-induced apoptosis could provide a valuable form of therapy.

## 2. UPR in Melanoma and Other Cancers

The UPR plays an important role in the function of cells and is routinely activated to deal with the high flux of proteins processed through the ER at certain times within the cell cycle. Cancers are subject to many forms of stress due to poor vascularisation and high proliferation. Therefore it is not surprising that the UPR is highly activated in cancer cells that are subject to hypoxia, nutrient deprivation and altered pH and require more proteins for neoplastic growth, in particular secretory proteins, to exploit their microenvironment. The UPR may assist in several aspects of tumour biology, ranging from tumourigenesis, apoptotic evasion, metastasis, angiogenesis and chemotherapy resistance. 

Numerous studies have found a link between activation of the UPR and cancer progression. One of the best studied proteins of the UPR is GRP78, levels of which are highly elevated in several cancers including prostate [41], colorectal [42,43], breast [44,45], ovarian and lung cancers [46]. In human melanoma samples, increased levels of GRP78 positively correlate with increased progression, tumour size and poor outcome for patients [47]. As such, elevated GRP78 has been identified as a potential biomarker for early diagnosis of melanoma [48]. Additionally, increased and sustained activation of other UPR mediators, IRE1 and ATF6 are critical for melanoma survival [49].

The UPR has been proposed as a critical early event in neoplastic transformation. In mouse models for breast and prostate cancer, GRP78 knock-out protects against cancer growth [50], proliferation and angiogenesis [51]. In human melanoma cells, knockdown of GRP78 results in decreased proliferation [52]. Similarly, decreases of other UPR mediators; XBP1, IRE1 and PERK, through knock-downs, knock-outs and null mutations in a range of cancer models result in decreased tumour size and reduced angiogenesis [53,54,55,56,57]. Additionally, paradoxically, auto-antibodies against GRP78 promote tumour growth and inhibit apoptosis by activating the UPR, resulting in growth and survival of melanoma, prostate and ovarian cancers [58,59,60].

Furthermore, the role of the UPR in cancer metastasis is becoming more evident, with research conducted into the contribution of ER stress and the UPR in cancer migration and invasion. Studies have focused in particular on dissecting the role of GRP78 in numerous human cancers. Elevated levels of GRP78 correlated with increased metastasis in prostate, gastric, colon, lung, esophageal and breast cancers; and hepatocellular and non-small cell lung carinomas *in vito* and *in vivo* [61,62,63,64,65,66,67,68]. In prostate, colorectal, gastric and breast cancer and esophageal squamous cell carcinoma, increased levels of GRP78 correlated with an increase in metastatic potential [61,62,65,66,68,69]. Comparison of primary cutaneous melanomas to their matched lymph node metastasis showed significant increases in GRP78 levels in disseminated melanoma. In primary cutaneous melanoma, Papalas *et al.* found a decrease in GRP78 with invasive depth but with a rapid increase of GRP78 levels at the invasive front of the tumour [70]. Studies in various cancer cell lines demonstrate the same positive correlation between increased levels of GRP78 with cell invasion and migration [63,64,65,66,67], with knockdowns of GRP78 *in vitro* result in decreased cell migration and invasion [63,64,65]. In addition to a decrease in metastastic potential, knockdown of GRP78 also resulted in the decrease of several proteins associated with metastasis, including vimentin, E-cadherin, MMP-2 (matrix-metalloprotease-2) and MMP-9 (matrix-metalloprotease-9) [64,65,67].

The increased metastatic potential with the UPR may in part be explained by its link to the epithelial to mesenchymal transition (EMT). Increased UPR, in particular the chaperone GRP78, has been found to promote EMT in various cell types including melanocytes and thereby promote tumourigenesis and dissemination. In breast cancer, the UPR mediator XBP1 was found to facilitate EMT (epithelial-to-mesenchymal transition) promoting tumour invasion [71]. EMT drives both neoplastic transformation and promotes a metastatic phenotype in melanoma with increased levels of EMT genes and EMT-inducing transcription factors conferring more adhesive, invasive and migratory properties [72,73]. 

In addition to the increased production of membrane and secretory proteins made possible by ER expansion and up-regulation of chaperones by the UPR, there are several other mechanisms that are of benefit to cancers. The UPR has been implicated in adapting the microevironment to the tumour’s needs, in other ways besides increasing secretory protein output. For example, the UPR can promote angiogenesis, essential for maintaining nutrient supply and growth for metastasis. GRP78 is found on the cell surface and secreted into the circulation by various solid tumours including melanoma [58,74,75]. Cell surface and circulating GRP78 has been found to act as a signaling hub and promotes cell proliferation and angiogenesis. One of the signaling responses induced by cell surface GRP78, that has strong implications in melanoma biology, is the upregulation of VEGF (vascular endothelial growth factor). VEGF stimulates the growth of solid tumours and angiogenesis in the tumour microenvironement. In melanoma patient samples, levels of VEGF correlate with cancer progression [76]. Karali *et al.* found that VEGF activated IRE1 and ATF6 through mTOR, contributing to the survival effect of VEGF on endothelial cells through activation of the Akt pathway. Furthermore, found that inhibition of several UPR mediators decreased VEGF-induced vascularisation in mouse Matrigel plug angiogenesis assay, comprising extracellular matrix proteins [77]. Cell surface GRP78 has also been shown to assist in invasion via its interaction with FAK (focal adhesion kinase), a major signaling protein in cell migration, adhesion and spreading. In hepatocellular carcinoma cell lines, over-expression of GRP78 caused an increase in FAK expression and tumour invasiveness [69]. Additionally, in colorectal cancer GRP78 increased cell migration and invasion into the EMC (extracellular matrix) through its interaction with β1-integrin and FAK [68]. 

Circulating GRP78 is capable of binding to endothelial cells and activating ERK and Akt signalling, protecting these cells from anti-angiogenic drugs (Figure 1) [78]. IRE1, PERK and ATF6 also directly regulate levels of VEGF mRNA [79]. The relationship between GRP78 and VEGF may be through reciprocal regulation, with Katanasaka *et al.* reporting increased GRP78 cell surface expression in VEGF-activated HUVEC (human umbilical vein endothelial cells) [80]. GRP78 has also been found up-regulated in tumour-associated macrophages that support tumour spread into the surrounding microenvironment [81].

It is evident that the role of the UPR is more widespread than previously thought, exemplified by research on GRP78. The influence of GRP78 on the EMT, its ability to stimulate angiogenesis via VEGF and to induce signaling cascades in neighbouring cells, suggests that the UPR repeals stress on a systemic level and has previously been oversimplified as a single cellular response. This widespread functionality may explain the paradoxical nature of GRP78, such as the ability of GRP78 auto-antibodies to perpetuate the UPR. Understanding how components of the UPR such as GRP78 promote tumour growth and metastasis through interaction with other cells and components of the tumour microenvironment is worthy of investigation. Additionally, therapies that target these specific interactions of the UPR with the tumour microenvironment may provide increased cancer specificity.

Another benefit of the UPR is its protein and organelle degradation mechanisms, ERAD and autophagy, respectively, the activations of which are coupled to the UPR. It has been proposed that these mechanisms play a key role during metastasis by recycling and supplying essential building blocks while the cell adapts to its new environment [82,83]. Recent evidence has implicated cellular dormancy in melanoma metastasis especially for uveal melanoma [84,85,86]. That has led to the proposal of prolonging melanoma dormancy as a possible treatment, with Ossowski and Aguirre-Ghiso proposing that therapies should focus on expanding long term dormancy [84]. A strong link has been established between tumour dormancy and the UPR, with ERAD proposed as an important stimulus in the growth of dormant metastases [82]. While mentioned briefly within this review, the roles of ERAD and autophagy in cancer are extensive and have been reviewed in detail elsewhere [25,82,87].

Research continues to unravel the effect of this complex stress response on cancer and to define the specific outcomes resulting from the UPR. For example, the effect of increased MEK/ERK signalling on tumourigenesis, the EMT switch promoting tumour dissemination and the effect of increased angiogenesis via VEGF for metastatic growth.

### UPR and MEK/ERK

One of the key oncogenic signaling pathways in melanoma is MEK/ERK [88], with BRAF, the upstream regulator, constitutively activated in 66% of malignant melanomas [89]. Oncogenic signaling from MEK/ERK increases cellular protein production thereby increasing the ER-burden and activating the UPR [52,90]. Indeed, it has been shown that MEK activation is essential for survival of melanoma under acute ER-stress [91]. Sustained induction of IRE1 and ATF6 is linked to increased MEK/ERK activation that protects melanoma from UPR-induced apoptosis, while inhibition of MEK/ERK partially blocks IRE1 and ATF6 [49]. It has also been reported that inhibition of BRAF or MEK prevents IRE1 and ATF6 activation, that in turn increases UPR-induced apoptosis [52]. Recent research suggests a reciprocal activation event between the UPR and MEK/ERK signaling that goes beyond a simple increase in cellular protein load, as stated above. Conversely, Beck *et al.* reported that melanomas treated with the RAF inhibitor vemurafenib had increased ER stress [92]. These contradictory findings may in part explain how melanoma adapts to chronic ER-stress. Constitutively active MEK may modulate particular arms of the UPR, such as IRE1, thereby preventing UPR-induced apoptosis while maintaining, or even increasing, its cytoprotective functions. 

## 3. UPR and Chemotherapy

### 3.1. Drug Resistance

The greatest challenge in the treatment of metastatic melanoma is its resistance to chemotherapy. Melanoma relatively quickly acquires resistance to drugs that are initially effective. Various studies have reported a correlation between increased levels of UPR markers and drug resistance [93]. Furthermore, the ER is a site for drug detoxification and the mere presence of anticancer drugs elicits an increased UPR response. In human melanoma cells, knockdown of GRP78 sensitised the cells to UPR-induced apoptosis under acute ER-stress, highlighting the potential of the UPR as a therapeutic target [52]. GRP78 has also been shown to protect against anti-angiogenic drugs in xenograft models of human breast cancers [78,94]. The UPR was found to be responsible for resistance of melanomas to vinca alkaloids, a class of anti-mitotic drugs that bind microtubules, used in combined therapies against metastatic melanoma [95]. Hypoxia is known to contribute to chemotherapeutic resistance through numerous mechanisms such as downregulation of DNA repair enzymes, poor drug delivery and chemical modification of drugs [96,97]. Under hypoxic conditions, the UPR is activated and initiates its cellular recovery program, allowing the cell to survive and adapt to the treatment. The hypoxia-sensitive protein Galectin-1 that is upregulated in melanoma, offers a cytoprotective effect to various anticancer drugs via modulation of the UPR in melanoma cells [98]. HDAC (histone deacetylase) is commonly up-regulated in cancer resulting in oncogenic activation by influencing both gene expression and direct modification of proteins. HDAC inhibitors directly influence aberrant gene expression via epigenetic regulation resulting in growth arrest and apoptosis in cancers. Despite showing promise as an anti-cancer therapy, either intrinsic or acquired resistance to HDAC inhibitors is commonly observed in sub-populations of cancer cells, with acquired cross-resistance to other anti-cancer drugs a major problem in this therapeutic strategy [99,100,101]. Numerous combinatorial therapies with HDAC inhibitors are currently under investigation in various cancers including melanoma. Inhibiting HDAC in melanoma cells improved the response to BRAF inhibitors, resulting in growth arrest and increased apoptosis [102,103]. HDAC inhibitors in combination with Ipilimumab, a monoclonal antibody against the immune suppressor CTLA-4, are currently in phase I trials for melanoma. HDAC1 is a repressor for GRP78 expression, inhibition therefore leads to increased UPR activation and resistance to HDAC inhibitors, while over-expression attenuates this resistance [104]. UPR inhibitors may overcome HDAC inhibitor resistance and are a potential avenue for combination treatment of metastatic melanoma. 

### 3.2. The UPR: As a Drug Target

The UPR is an attractive therapeutic target due to its link with apoptosis and role in drug resistance. Harnessing apoptosis mechanisms and inhibiting pathways that evoke resistance is a current focus for anticancer drug development (Table 1). The UPR is up-regulated in cancers providing a means by which drugs can be targeted specifically to cancer cells through targeting UPR mediators. One of the main difficulties in finding effective treatments for cancer is establishing a therapy that is effective given the heterogeneity of malignancies, among sub-clone metastases from a single tumour. The UPR is not inherently an oncogenic pathway; rather, it is a normal cellular process that may be corrupted for the benefit of the cancer. Therefore, targeting the UPR could be effective against a wide range of cancers despite their individual mutational status. The UPR, specifically GRP78, assists in angiogenesis, therefore inhibiting the UPR may block both UPR associated angiogenesis and cytoprotection. Additionally, the UPR up-regulates numerous pro-apoptotic proteins that initiate several apoptotic cascades. Of particular interest for the treatment of melanoma, which is notoriously resistant to apoptosis, is the direct activation of caspase-3 via JNK.

A number of drugs directly targeting the UPR are currently in clinical trial, including several GRP78 inhibitors (Table 1). PAT-SM6, in Phase I clinical trials against melanoma and Phase I/ IIa in multiple myeloma, is a monoclonal antibody reported to bind a cancer specific GRP78 cell surface isoform, thereby inducing apoptosis in cancer cells [105,106]. Another GRP78 targeting drug is DHA (docosahexaenoic acid), an omega-3 fatty acid, that inhibits total and cell surface GRP78 expression and increases apoptosis in cancer cells [107,108,109]. In melanoma cell lines, DHA induces cell cycle arrest and increased apoptosis [110]. As DHA is not only non-toxic but actually carries health benefits, its positive effects have been widely tested on numerous cancers, showing decreased growth and metastasis [109,111,112,113]. Under the stress of nutrient-deprivation, Arctigenin, a plant lignin, specifically blocked the expression of GRP78 with activation of XBP1 and ATF4, resulting in ROS/MAPK-mediated apoptosis [114,115,116]. The UPR mediator PERK, that suppresses global protein synthesis, controls ATF4 transcriptional regulation of UPR responsive genes and CHOP-mediated apoptosis is another major target for drug development. Small molecule drug screening has identified several PERK inhibitors, including GSK2656157 and GSK2606414, that exhibited anti-tumoural effects but with severe side-effects against pancreatic tissue [117,118]. Indeed, cells that have a functionally high secretory protein burden and therefore constant induction of the UPR, such as pancreatic β-cells, have been identified as a major obstacle to targeting the UPR. As such, the rationality of targeting major UPR components, such as GRP78, must be questioned and the implications of therapeutics directed at the source of this widespread and uncharacterized response examined, especially given the contradictory role exhibited by GRP78. Greater therapeutic benefit may be gained by targeting multiple downstream effectors and UPR-tumour specific interactions. 

Numerous studies have reported increased efficacy of existing chemotherapies when combined with both UPR inhibitors and activators, to prevent cytoprotective effects or induce apoptosis. Furthermore, the co-activation of MEK/ERK and the UPR provides an interesting opportunity for combination therapies in melanoma targeting this key oncogenic pathway. Patients with late stage BRAF mutant melanomas administered vemurafenib, a BRAF inhibitor, show significant tumour regression and increased survival. However, relatively rapid resistance is acquired, with most patients relapsing with a lethal drug resistant phenotype. Interestingly, induction of ER-stress and the UPR in vemurafenib-resistance melanoma results in increased apoptosis [92]. In a melanoma mouse model, Thakur *et al.* showed that proliferation of vemurafenib-resistant cells was dependent on the presence of the drug [123]. A combination therapy alternating vemurafenib and UPR inducers may prevent the emergence of drug resistance. The GRP78 suppressor, Arctigenin, mentioned above, sensitises cancer cells to cisplatin-induced apoptosis via STAT3 inhibition [124]. DHA, an omega-3 fatty acid, sensitises cancers to various chemotherapies [125,126,127]. Phase 3 clinical trials for metastatic melanoma have been conducted with DHA conjugated to paclitaxel, a microtubule disrupting agent. Despite limited patient benefit, the drug was well-tolerated leading to speculation that further combined therapies with DHA-paclitaxel may have increased efficacy [121,122]. In melanoma cell lines DHA has exhibited synergy with cyclooxygenase inhibitors and decreased melanoma growth with type 1 transforming growth factor beta [128,129]. The chelating agent, D-penicillamine, currently used to treat rheumatoid arthritis, was found to induce caspase-dependent apoptosis in cultured metastatic melanoma cells with activation of the UPR [130]. Inhibition of the UPR also increased the efficacy of DNA-damaging agents, such as cisplatin and adriamycin in human melanoma cells. Both cisplatin and adriamycin increased the UPR, and silencing GRP78 sensitised melanoma to apoptosis induced by these agents [131]. Bortezomib, an inhibitor of the 26S proteosome, that induces the accumulation of unfolded proteins, is effective against a range of cancers by increasing cellular stress and initiating UPR-induced apoptosis. In melanoma, bortezomib was found to induce ER stress and increase apoptosis [132,133]. Bortezomib was also found to inhibit nuclear factor κB-mediated gene expression in melanomas *in vitro*. The combination of Bortezomib with temozolomide in melanoma mouse models induced long-term remission [134]. Melanoma progression correlates with decreased expression of TRAIL (tumour necrosis factor-related apoptosis-inducing ligand) receptors, with most cancers resistant to TRAIL-induced apoptosis [62]. The UPR has been found to increase TRAIL-induced apoptosis in melanoma cells via up-regulation of the TRAIL receptor [135]. Drug synergy between bortezomib and TRAIL has been demonstrated in melanoma cells by increasing TRAIL-induced apoptosis [136,137]. When used in combination with other drugs the effects were even more potent, as seen in melanoma treated with bortezomib, TRAIL and a SMAC (second mitochondria-derived activator of caspase) mimetic [138]. Fenretinide, a retinoic acid derivative that produces reactive oxygen species and increases levels of unfolded proteins, also induced the UPR in melanoma. Furthermore, inhibition of GRP78 and PDI (protein disulfide isomerase), another UPR mediator, sensitised melanoma to apoptosis in combination with bortezomib and fenretinide [139,140,141]. Additionally of importance in the treatment of metastatic melanoma is the contribution of the UPR to vemurafenib-resistance. Patients with late-stage BRAF mutant melanomas administered vemurafenib, the BRAF inhibitor mentioned above, show significant tumour regression and increased survival [142,143]. However, relatively rapid resistant is acquired with most patients relapsing with a lethal drug resistant phenotype [144]. Induction of ER-stress and the UPR in vemurafenib-resistance results in increased apoptosis [92,145]. This provides a potential therapeutic combination. 

The reliance of cancer on the UPR and the increased levels of UPR mediators provides a target for development of anti-cancer drugs. Prasad *et al.* reported that activation of the UPR in melanoma, specifically IRE1 and XBP1, prior to the introduction of oncolytic viruses enhanced adenovirus levels specifically in cancerous cells and resulted in increased tumour cell killing [146]. In addition, up-regulated GRP78 on the surface of cells is a target for peptidic ligands to melanoma [147], a potential avenue for specific peptide-conjugated drug delivery to cancers.

## 4. Conclusions 

The role of the UPR in cancer progression and metastasis, particular for melanoma, continues to grow. The link between melanoma progression and the UPR is well established, what is less clear is how cancers adapt to chronic UPR induction while being resistant to its apoptotic mechanisms. Understanding the factors that influence the balance between the survival and death responses of the UPR is essential for targeting this stress response. Given that cancers constantly activate the UPR to respond to environmental stress, it is likely that they develop mechanisms to evade UPR-induced apoptosis. Therefore, combinatorial drug treatments that target the UPR are promising with dual treatments expected to be most effective in combating melanoma. Further research into the role of the UPR in malignancy will help in development of drugs that modulate drug resistance and apoptosis. The dependence of melanomas on the UPR for survival has prompted focused interest on this pathway for development of effective treatments for metastatic melanoma. 

## Figures and Tables

**Table 1:** Drug therapies targeting the UPR for the treatment of cancer. Superscript denotes combinatorial therapeutic trials.

Drug/s	Target	Effects	Study /clinical trial
Versipelostatin	GRP78 and GRP94	Inhibits transcription of GRP78/94 target genes Initiates UPR-induced apoptosis under glucose deprivation	Preclinical [119,120]
Docosahexaenoic acid	GRP78	Decreased levels of GRP78 Induced apoptosis Increased expression of UPR proteins ERdj5 and PERK	Preclinical **melanoma** [110] Phase II/ III **melanoma** [121,122] Phase II/III/IV solid tumours
PAT-SM6	GRP78	Monoclonal antibody binds cell surface GRP78 to induce apoptosis	Phase I **melanoma** [105] PhaseI/ II multiple myeloma
Arctigenin	GRP78	Induces apoptosis via ROS/MAPK	Preclinical [114,115,116]
Bortezomib *in combination with* ^1^*azacytidine,* ^2^*decitabine*	26S proteosome	Inhibits ERAD Increases ER-stress from accumulated misfolded proteins Induces UPR-mediated apoptosis	FDA-approved multiple myeloma, acute myeloid leukemia, Phase II metastatic melanoma ^1,2^Phase I multiple myeloma
Carfilzomib	26S proteosome	Inhibits ERAD Increases ER-stress from accumulated misfolded proteins Induces UPR-mediated apoptosis	Phase III multiple myeloma Phase II lymphoma
GSK2656157	PERK	Inhibits PERK kinase Decreases blood vessel density	Preclinical [117,118]
ISRIB	ATF4	Inhibits ATF4 expression Reverses eIF2α effects	Preclinical
